# The Metano Modeling Toolbox MMTB: An Intuitive, Web-Based Toolbox Introduced by Two Use Cases

**DOI:** 10.3390/metabo11020113

**Published:** 2021-02-17

**Authors:** Julia Koblitz, Sabine Eva Will, S. Alexander Riemer, Thomas Ulas, Meina Neumann-Schaal, Dietmar Schomburg

**Affiliations:** 1 Leibniz Institute DSMZ-German Collection of Microorganisms and Cell Cultures, Inhoffenstraße 7 B, 38124 Braunschweig, Germany; julia.koblitz@dsmz.de (J.K.); sabinewill@outlook.de (S.E.W.); 2 Department of Bioinformatics and Biochemistry and Braunschweig Integrated Center of Systems Biology (BRICS), Technische Universität Braunschweig, Rebenring 56, 38106 Braunschweig, Germany; alex_riemer@gmx.de (S.A.R.); t.ulas@uni-bonn.de (T.U.)

**Keywords:** systems biology, metabolic modeling, flux balance analysis, *Phaeobacter inhibens*, tropodithietic acid

## Abstract

Genome-scale metabolic models are of high interest in a number of different research fields. Flux balance analysis (FBA) and other mathematical methods allow the prediction of the steady-state behavior of metabolic networks under different environmental conditions. However, many existing applications for flux optimizations do not provide a metabolite-centric view on fluxes. Metano is a standalone, open-source toolbox for the analysis and refinement of metabolic models. While flux distributions in metabolic networks are predominantly analyzed from a reaction-centric point of view, the Metano methods of split-ratio analysis and metabolite flux minimization also allow a metabolite-centric view on flux distributions. In addition, we present MMTB (Metano Modeling Toolbox), a web-based toolbox for metabolic modeling including a user-friendly interface to Metano methods. MMTB assists during bottom-up construction of metabolic models by integrating reaction and enzymatic annotation data from different databases. Furthermore, MMTB is especially designed for non-experienced users by providing an intuitive interface to the most commonly used modeling methods and offering novel visualizations. Additionally, MMTB allows users to upload their models, which can in turn be explored and analyzed by the community. We introduce MMTB by two use cases, involving a published model of *Corynebacterium glutamicum* and a newly created model of *Phaeobacter inhibens*.

## 1. Introduction

Genome-scale metabolic models are important tools for systems biology. They are used in various fields, e.g. in metabolic engineering, the identification of potential drug targets, and as knowledge libraries to understand the behavior of biological systems in detail [1]. In these applications, mathematical methods are essential for the calculation of flux distributions in large networks.

Flux balance analysis (FBA) allows the prediction of the steady-state condition of the metabolic network of an organism under different environmental conditions [2]. Metabolic networks typically have more reactions than metabolites, which results in an under-determined solution space. The standard approach to solve the under-determined system is to apply linear programming. In this approach, constraints are added to reduce the solution space. The optimal solution is one that maximizes or minimizes a given objective function, e.g. biomass production. The underlying assumption is that the organism has been optimized through evolution for a biological goal, such as optimal growth or minimal nutrient uptake [3]. In contrast, minimization of metabolic adjustment (MOMA) is used to calculate the flux distribution for perturbed networks, such as in knock-out mutants [4]. Due to the perturbation of the network, the assumption of FBA that the organism is evolved for a biological goal may not be valid. The underlying assumption of MOMA is that the organism undergoes a minimal redistribution of the network. For this reason, MOMA employs quadratic programming to find a solution that is closest to the wild-type flux distribution. Flux variability analysis (FVA) is an approach for analyzing the robustness of a metabolic network [5]. FVA is used to find the minimal and maximal flux for each reaction in the network while constraining some states of the flux distribution, e.g. limiting the biomass production flux to at least 95% of the FBA maximum.

As most applications for flux optimization focus on a reaction-centric point of view, they pay little attention to metabolite fluxes. Only a small number of previous studies showed the benefit of analyzing models from a metabolite-centric point of view, e.g. for the discovery of new drugs [6,7]. There are also algorithms that maximize specific metabolite fluxes for the calculation of metabolic changes in order to predict the effect of certain drugs [8]. However, these methods were never integrated into a comprehensive modeling toolbox and can only be reproduced by experts in programming and metabolic modeling. 

Metabolic modeling is used for a variety of applications, ranging from biotechnological applications to ecological questions [9]. A well-characterized marine model organism is *Phaeobacter inhibens* DSM 17395, an ideal model organism for basic research and elucidation of stress responses because of its metabolic versatility. Experimental data for degradation pathways of different carbon sources based on metabolome and proteome measurements have been determined for *P. inhibens* DSM 17395 in previous studies by us and others [10,11,12,13,14]. The organism carries three extrachromosomal elements (65 kb, 78 kb and 262 kb) [15,16]. Proteins required for the biosynthesis of the antibacterial compound tropodithietic acid (TDA) are encoded on one of them [15,17,18]. Deletion of the plasmids leads to large variations in the growth efficiency with amino acids as a carbon source [19]. More recently, we could show that the main reason for higher growth yields of the Δ262-kb plasmid-cured mutant strain are higher non-growth-associated energy requirements of the wild-type strain because its antibiotic TDA disturbs the membrane proton gradient [20,21]. Thus, wild-type and TDA-negative mutant strains of *P. inhibens* DSM 17395 are applicable for metabolic modeling. 

The second model system of this study is *Corynebacterium glutamicum* DSM 20300, a widely used biotechnological production strain that was originally isolated as an l-glutamate-producing bacterium [22] and is used to produce about 2.5 million tons of glutamate per year [23]. Furthermore, *C. glutamicum* has been metabolically engineered for the production of other amino acids, organic acids, alcohols, and polymers [23]. 

In this study, we present Metano, an open-source command-line toolbox for metabolic modeling, and MMTB, a web-based interface to Metano that comes with an integrative database of biochemical reactions and genome annotations. While Metano was developed for a more versed audience, MMTB was designed especially for inexperienced users or users without a strong background in bioinformatics. 

We applied Metano methods to the published model of *C. glutamicum* to verify the accuracy or capability of the toolbox. In a second step, we used the TDA-negative mutant strains of *P. inhibens* DSM 17395, with optimal growth on three different amino acids to reconstruct a metabolic model for *P. inhibens* DSM 17395, based on genomic and experimental data. FBA was performed to analyze the carbon flux in the wild-type strain compared to the TDA-negative mutant strains with respect to carbon loss and growth inhibition by TDA. MMTB methods provided a metabolite-centric view on the model to determine changes in the intracellular carbon flux under different growth conditions.

## 2. Results

### 2.1. Metano Standalone Toolbox

Metano is a Python-based command-line tool for the analysis of stoichiometric models. Several features can be accessed online via the MMTB. Prominent examples of constraint-based metabolic modeling software include COBRA [24], FASIMU [25], OptFlux [26], and FAME [27]. The freely available COBRA Toolbox [24] includes a continuously expanding range of algorithms for flux optimization and metabolic engineering. COBRA is a MATLAB toolbox, which also provides interfaces in Python [28] and other languages. Being the most popular tool in this research field, we chose the COBRA toolbox for comparison (Table 1). The command-line oriented software FASIMU [25] also has broad functionality. It offers batch computation and introduces concentration-based thermodynamic feasibility as a constraint. OptFlux [26] offers a graphical user interface, a broad range of features, and many metabolic engineering tools, which makes it better suited for biotechnologists than the other toolboxes. FAME [27] is an example of an exclusively web-based software solution. It allows the user to create, edit, and run metabolic models in an online application. FAME also supplies visualization of analysis results on KEGG-like maps.

While flux distributions in metabolic networks are conventionally analyzed from a reaction-centric point of view, the Metano methods of split-ratio analysis and metabolite flux minimization (MFM) allow a metabolite-centric view on flux distributions [29]. Split-ratio analysis gives insight into all reactions that are involved in the production or consumption of a specific metabolite. MFM is a useful tool for the determination of the essentiality of metabolites for optimal biomass production and flux distributions at branch points. Additionally, a ranking of all metabolites by their minimal flux can give further insights into the behavior of the metabolic network and the role of each metabolite within. Metano also supports dynamic network visualization, utilizing the optional add-on AMEBA [29]. This tool provides an interactive graphical user interface, which allows the user to navigate through the metabolic network by clicking on different nodes in the bipartite reaction graph. AMEBA provides an integrated metabolite-centric view with split-ratios of each metabolite that can be used for the refinement of genome-scale metabolic models [29]. 

The Metano toolbox includes several features for flux optimization and is optimized for processing speed. In addition to the common FBA algorithm, Metano implements the fastFVA algorithm for FVA [30]. Metabolic flux minimization (MFM) is very similar to FVA and is also amenable to the fastFVA strategy, but in a metabolite-centric approach. MOMA has been implemented with the option to perform an FVA-based reduction of the solution space as a preprocessing step, which can speed up the analysis significantly. In the implementations of all the above-mentioned algorithms, iterative dead-end analysis is performed prior to the respective optimization problem. This is done to reduce the dimension of the solution space, hence reducing the calculation time substantially. Due to its efficient run-time, Metano has been applied to large-scale analyses involving hundreds of thousands of simulations [31,32,33,34,35,36]. The toolbox was proven to be able to handle larger models with more than one thousand reactions and multiple compartments, e.g. the iND750 model of Saccharomyces cerevisiae [37]. 

In addition to the widely used SBML format, Metano models can be represented by simple lists of reactions that are readable by humans and can be easily modified by the user with any text editor. An additional parameter file sets the constraints for a simulation scenario so that the variables, e.g., substrate boundaries and objective function, are detached from the model itself. Easy exchange of models is ensured by offering export and import tools for the widely accepted standard format SBML [38].

### 2.2. MMTB Website

Metano Modeling Toolbox (MMTB) is a web-based tool for the generation and analysis of metabolic models. MMTB closes the gap between BKMS-react [39] and EnzymeDetector [40,41]: biochemical reactions from BKMS-react are associated with genome annotations from EnzymeDetector and preprocessed for use in metabolic modeling. Information on metabolite structures and synonyms and the assembly of reactions into pathways support bottom-up model generation. Additionally, MMTB allows OS-independent analyses and visualization of metabolic models: an interface to the modeling toolbox Metano provides different analysis methods and a metabolite-centric view of networks.

**Figure 1:**
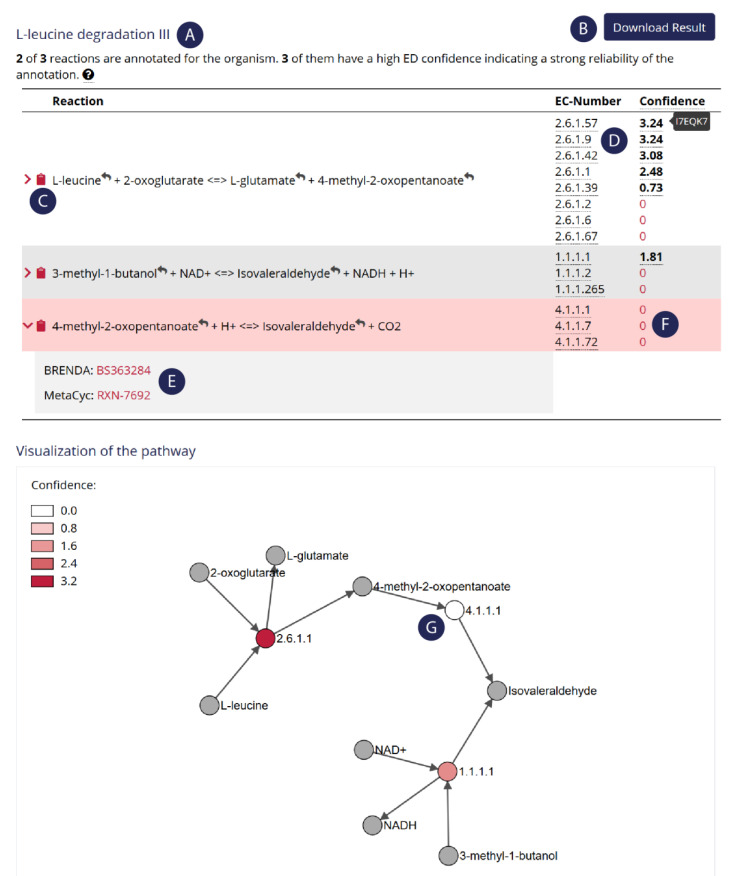
Screenshot of the output of a pathway search on MMTB. The selected pathway was l-leucine degradation (from MetaCyc) and the selected organism was Phaeobacter inhibens DSM 17395. MMTB informs about annotated enzymes from this pathway (**A**). Users can either download the whole pathway (**B**) or copy single reactions to the clipboard (**C**). MMTB shows annotation confidence scores for each enzyme that catalyzes this reaction, as well as the corresponding UniProt accession on mouse over (**D**). Reactions are cross-linked to other databases (**E**). Missing enzymes are highlighted in the reaction table (**F**). The color code in the bipartite graph also indicates the presence and absence (**G**) of enzymes, suggesting that this certain pathway is not present in the selected organism.

On the one hand, MMTB assists during model creation. The key feature is an intuitive search tool that gives access to reaction and metabolite information. The user can search for reactions by either entering parts of the biochemical reaction, EC numbers, database IDs, or by selecting a BRENDA, MetaCyc, or KEGG pathway. The resulting reactions originate from the integrative database BKMS-react [39] that includes all reactions from BRENDA [41], KEGG [42], MetaCyc [43], and SABIO-RK [44]. MMTB shows ready-to-use reactions and further information, e.g., the assigned EC numbers, database IDs, associated pathways, and further links. Selected pathways are further visualized as a bipartite graph (Figure 1). When the user selects an organism from a dropdown list, MMTB displays a confidence score for the annotation of each enzymatic reaction. This score comes from EnzymeDetector, which integrates genome annotations from different resources [41]. All reactions can be downloaded in a plain reaction file for use with the Metano command-line tool. Additionally, the user can search for metabolite synonyms and receive information on recommended names, synonyms, molecular weight, and the chemical structure. Metabolites can also be identified by using regular expressions. 

On the other hand, MMTB assists during model analysis via an interface to the Metano toolbox. The user can upload a metabolic model and apply one of the following analysis methods: (1) FBA, (2) FVA, (3) dead-end analysis, (4) split-ratio analysis, (5) metabolite flux minimization, (6) a metabolite-centric visualization that shows the network as an interactive bipartite graph with consuming and producing metabolite fluxes, (7) a model verification tool where all metabolites in the uploaded model are replaced by their recommended names from BKMS-react. Because misspelled metabolites or synonyms are not treated as identical metabolic nodes, this unique method helps to avoid mistakes. 

Additionally, MMTB has a model exchange platform, where users can upload and publish their own models. These models can be viewed, analyzed, visualized, and downloaded, thus promoting the reuse of models that are already published. A model conversion tool for SBML and JSON models allows the use of models that were generated with other software. 

### 2.3. The Metabolic Model of Corynebacterium Glutamicum

The Metano modeling toolbox was evaluated using the published *C. glutamicum* model iMG481 that consists of 550 reactions [45]. The online conversion tool of MMTB was used to convert the model from SBML into the human-readable Metano plain text format. The model was then uploaded to the MMTB modeling platform.

By using the Metano toolbox, we were able to reproduce the in silico growth on glucose as it was described by Graf and colleagues. When using maximal biomass production as objective function, Metano was able to reproduce the growth rate of 0.337 h^−1^, as well as key fluxes described in the paper. Furthermore, the MFM method revealed that of a total of 408 metabolites, 195 were not essential for biomass production of 95% of the optimal value. When the biomass function is restricted to 10%, 205 metabolites are non-essential. 

### 2.4. The Metabolic Model iPin571 and Biological Implications

The model iPin571 was manually assembled with the assistance of the MMTB database of metabolic reactions and the integrated EnzymeDetector annotation (Supplementary Table S1). The model was published under Creative Commons Attribution License and is available on the MMTB modeling platform. The model consists of 486 genes, 571 metabolic reactions (13 not sequence-based, 7 spontaneous), 35 transport reactions, 22 exchange reactions, and 564 metabolites. The biomass reaction was divided into 17 reactions overall, which allows an easy adaptation of the model to change biomass compositions. The model includes all necessary biosynthesis pathways and energy metabolism. Additionally, degradation pathways of amino acids, carbohydrates, and certain further carbon sources were implemented based on experimentally determined metabolome and proteome data [10,11,12,13,14]. TDA biosynthesis is represented by an overall reaction, starting from the phenylalanine degradation product 3-oxo-5,6-dehydrosuberyl-CoA semialdehyde, as the details of the biosynthesis pathway are still not fully understood [17,18,46]. 

Nine of the proteinogenic amino acids were previously analyzed with respect to growth physiology and degradation pathways [10]. For the analysis of the influence of TDA production on the metabolism, three different amino acids were chosen: (1) l-phenylalanine as the precursor for TDA biosynthesis, (2) l-alanine as an amino acid with immediate access to the central carbon metabolism, and (3) l-leucine as an amino acid with a more complex degradation pathway, slower growth, and a reduced TDA production compared to l-alanine. TDA-negative strains (Δ262-kb plasmid-cured and tdaE transposon mutant strains) were cultivated as a reference to compare optimal growth with inhibited growth, and furthermore, to analyze the influence of carbon loss due to secreted metabolites. 

The model iPin571 was able to simulate growth for the TDA-negative mutants on different carbon sources and the wild-type strain on leucine (Table 2). 

The wild-type strain showed the lowest growth yields on each of the amino acids as a nutrient, especially on l-phenylalanine, indicating reduced carbon yields due to TDA biosynthesis (Supplementary Table S2). Growth yields of about 11–15 g mol_C_^−1^ were reached with TDA-negative mutant strains, similar to the growth yield of the Δ262-kb plasmid-cured mutant strain with casamino acids as carbon source [19]. The growth yield of the Δ262-kb mutant strain was about 1.7-fold (l-alanine), 1.3-fold (l-leucine), and 1.6-fold (l-phenylalanine) higher compared to that of the wild-type strain. The tdaE transposon mutant strain showed almost the same growth yields as the Δ262-kb mutant strain, indicating that the lower growth yields of the wild-type strain were connected to the inhibitory effect of TDA with a necessity for an increased respiratory activity resulting in more carbon loss by CO_2_ production [20,21].

The model was used to predict the carbon loss caused by TDA production. In absence of TDA production, growth rates of P. inhibens DSM 17395 varied with the three amino acids due to different specific uptake rates (Table 2). However, with active TDA biosynthesis, specific uptake rates were higher, correlating with lower biomass yields. Without TDA production, about 50% of the carbon was used for biomass production and about 50% was respired to CO_2_ (Table 3). 

The wild-type flux distribution was similar to the mutant only when growing on l-leucine as a carbon source, because very low amounts of TDA are produced under this condition. With l-alanine and l-phenylalanine, only 32–34% of the available carbon was used for biomass production, while 15–19% of the available carbon was used for TDA production. However, due to the inhibitory effect by degrading the proton gradient [20,21], a higher CO_2_ production could be possible.

**Figure 2:**
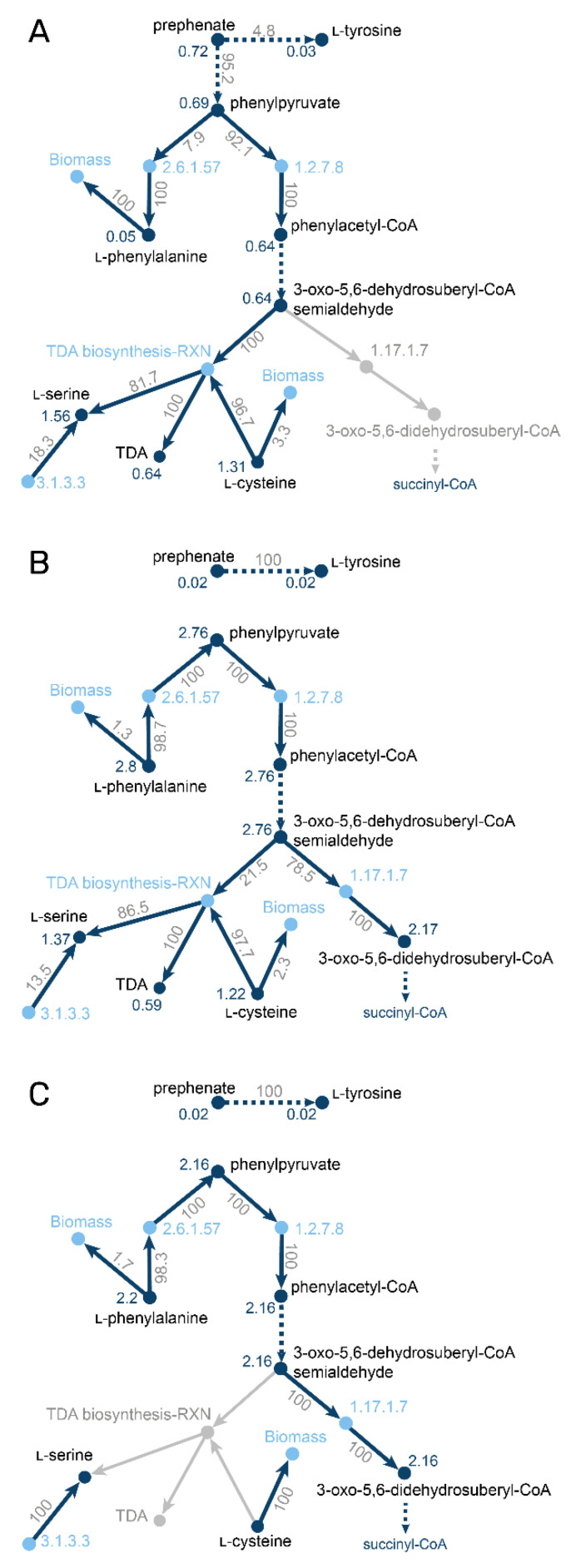
Metabolite-centered visualization of split-ratio analyses of the branch point between phenylalanine degradation and TDA biosynthesis. Reaction nodes are shown in light blue and labelled with the E.C. number if available. Metabolite nodes are shown in dark blue and the blue labels at the metabolite nodes are absolute metabolite fluxes in mmol g CDW^−1^ h^−1^. Edge labels between substrate and enzyme refer to relative fluxes outcoming from the metabolite, labels between enzyme and products refer to relative incoming fluxes of the metabolite. Grey parts of the network are inactive under the shown condition. Multiple reactions without branch points were condensed into dark blue dashed arrows. (**A**) simulation of wild type growth on l-alanine; (**B**) simulation of wild type growth on l-phenylalanine; (**C**) simulation of the *tdaE* mutant on l-phenylalanine.

We used the metabolite-centric view of MMTB to analyze the different growth behaviors of the wild type. The analysis showed that while growing on l-phenylalanine, 21.5% of the carbon source is converted into TDA (Figure 2). When growing on l-alanine, 0.72 mmol g_CDW_^−1^ h^−1^ of prephenate, a precursor of phenylalanine, is synthesized. Only 8% of prephenate is further converted to l-phenylalanine, while 92% is used to produce TDA. The total amount of produced TDA is similar under both conditions. In contrast, when using l-leucine as a carbon source, no TDA is produced, resulting in a total flux through prephenate of 0.03 mmol g_CDW_^−1^ h^−1^ and a higher carbon yield compared to the other conditions.

## 3. Discussion

In this study, we presented Metano, a standalone toolbox for metabolic modeling. Metano provides a large number of analysis tools, is easily extensible, and supports different model formats. The toolbox includes several computationally efficient analysis methods, an interactive GUI for network visualization, a module for batch FBA simulation, and a metabolite-centric view on flux distributions that is the first of its kind. The Metano method of metabolite flux minimization uses a variability analysis approach to minimize the fluxes through metabolite nodes. While other studies already pointed out the importance of metabolite fluxes, e.g., for drug discovery, we implemented this approach in a comprehensive toolbox. 

Metano is extended by MMTB, a web-based toolbox for metabolic modeling. MMTB integrates EnzymeDetector annotations and biochemical reactions from different databases to assist during model creation. Web-based analyses, for example, FBA, FVA, MFM, and split-ratio analysis, allow users to analyze models without a local installation. An intuitive user interface introduces inexperienced users to stoichiometric modeling. In addition, a modeling platform drives the reuse of models and guides new users through the wide landscape of existing models. Conversion tools for SBML and JSON models further support this aspect. In contrast to well-established toolboxes, such as COBRA that provide many advanced methods especially for metabolic engineering, MMTB focuses on usability and a less experienced audience that want to effortlessly explore metabolic modeling, without refraining from the accuracy or capability of a full-fledged modeling toolbox. We confirmed the latter by applying Metano methods to the model iMG481 and comparing the results with published analyses.

Moreover, we applied Metano methods to the published model of *C. glutamicum* DSM 20300. We were able to evaluate the accuracy and reliability of Metano methods by reproducing results from the primary literature. Furthermore, we applied Metano methods to create and elucidate a metabolic model of *P. inhibens* DSM 17395. We were able to simulate both the metabolic versatility and the growth-inhibiting effect of TDA. Furthermore, metabolite-centric analyses and visualizations highlighted metabolic fluxes that contribute to carbon loss and growth inhibition. 

## 4. Materials and Methods 

### 4.1. Strains and Growth Conditions

In this study, *P. inhibens* DSM 17395, its plasmid-cured derivative strain Δ262-kb [19] and the transposon mutant strain *tdaE* [20] were used. All cultivations were performed in a defined saltwater medium [14] with single amino acids as C-equivalent, sole carbon source: 30 mM l-alanine, 10 mM l-phenylalanine, or 15 mM l-leucine. 

Cultivations were started in 20 mL saltwater medium containing 1% casamino acids (Merck, Darmstadt, Germany), and cells were transferred at least for one adaptation cycle in the respective amino acid before inoculating the main cultures. All main experiments were conducted with three biological replicates in 500 mL Erlenmeyer flasks with three baffles at 150 rpm and 28 °C in an orbital incubator as previously described [20].

To make sure that oxygen is not limited, dissolved oxygen was measured with an online oxygen monitoring device (Shake Flask Reader; PreSens, Regensburg, Germany) as previously described [20]. For determination of the biomass composition, six biological replicates of each strain were cultivated with each single amino acid. Growing cells were harvested and centrifuged at 4 °C and 12,000× *g* for 5 min. Cells were re-suspended in 3.7% sodium chloride and split to get 1–3 mg cell dry weight (CDW) for DNA, RNA, protein and polyhydroxybutyrate (PHB) each, 10–30 mg for lipid and 10–30 mg for determination of CDW (Supplementary Tables S3 and S4).

### 4.2. The Metabolic Model iMG481

The metabolic model *i*MG481 was obtained from the Supplemental Material of Graf and colleagues [45]. The model is a modified version of iEZ475 [47], that is in turn a modified version of iKK446 [48]. It was converted into the Metano reaction format using the SBML conversion tool of MMTB. The model was furthermore distributed by uploading it to the MMTB model platform under the terms of the Creative Commons Attribution License (CC-BY). FBA was performed using maximum biomass yield as objective function.

### 4.3. Reconstruction of the Metabolic Model iPin571

The metabolic model *i*Pin571 was reconstructed based on genome and experimental data [10,11,12,13,14,15,17,18,46]. The web tool MMTB presented here was used for model reconstruction and validation. The model was built bottom-up using the pathway search of MMTB. All pathways for biomass production and degradation of various carbon sources were checked using the built-in genome annotation score from EnzymeDetector [41]. We included all necessary pathways into the model and matched gene locus tags using the UniProt accessions that were given by MMTB (Supplementary Table S1). The biomass composition was experimentally determined (Supplementary Tables S3 and S4) and added as biomass reaction for each strain and carbon source in the model. Flux balance analyses (FBA) were performed with the presented Metano toolbox. For analysis of the carbon flux distribution, the upper bound of the biomass reaction flux was set to the experimentally determined growth rate. FBA was performed using maximum biomass yield as objective function.

### 4.4. Algorithm

Metano is a software toolbox for metabolic modeling implemented in the Python programming language. Metano is easily extensible by prospective functional extensions via well-documented interfaces in a structured object-oriented framework. This allows the adaptation of the software to different requirements by users with basic programming skills. Additionally, all functionalities of Metano can be accessed by standalone Python programs via the command line, without the need to write programs. 

The Metano toolbox implements many well-established analysis tools, including FBA, FVA, MOMA, knockout simulation, and dead-end analysis [49]. Advanced features include tools for split-ratio analysis and metabolite flux minimization (MFM) [29], FBA batch computation, a prediction tool for directionality from Gibbs free energies, automated plausibility checking of metabolic models, and the generation of comparative scatter plots. 

MFM first performs a standard FBA and then adds a variability analysis comparable to FVA by the following algorithm: (1) Set a constraint for the objective function (default 95% of FBA flux). (2) Generate coefficient vectors expressing the (producing) metabolite fluxes as linear combinations of the reaction fluxes. (3) Perform linear programming with the linear objective functions defined by these coefficient vectors to successively minimize each objective function.

Split-ratio analysis computes the split ratios for the fluxes entering and leaving each metabolite as totals fluxes and percentage values. This method is used to calculate the network and label edges and nodes for the visualization available at MMTB.

All methods are described in more detail on the software website and in the Supplementary Appendix. Additionally, the documented source code is available online: github.com/JuliaHelmecke/metano (accessed on 9 Febraury 2021).

Metano includes interfaces to numerous freely available solvers for mathematical optimization problems. For linear programming, the supported solvers are lp-solve and the GNU Linear Programming Kit (GLPK), interfaced to Python via Swiglpk and PyMathProg, respectively. Quadratic programming problems are solved by using the quadratic cone program solver of CVXOPT, interfaced via CVXPY.

The software is published under the GNU General Public License version 3 and is available via the MMTB website (mmtb.brenda-enzymes.org (accessed on 9 Febraury 2021)). Metano does not rely on commercial software packages and can be installed on various Linux distributions via the Python Package Index (PyPI) and the related package installation system pip. A current version of Metano is also available for OS-independent use as a Docker image on Docker Hub.

## Figures and Tables

**Table 1:** Feature comparison between Metano and existing software. Divided by horizontal lines are different sections: functionality, usability, visualization, exchangeability, compatibility, and assistance in model reconstruction. FBA: flux balance analysis; FVA: flux variability analysis; MOMA: minimization of metabolic adjustment; ROOM: regulatory on-off minimization; MFM: metabolite flux minimization; GUI: graphical user interface.

Feature	Metano	COBRA	FASIMU	OptFlux	FAME
FBA	+	+	+	+	+
FVA	+	+	+	+	+
MOMA	+	+	+	+	-
ROOM	-	-	+	+	-
MFM	+	-	-	-	-
Batch computation	+	-	+	-	-
OptKnock	-	+	-	+	-
Command line	+	+	+	-	-
GUI for modeling	-	-	-	+	-
Independence of commercial software	+	-	+	+	+
Dynamic visualization GUI	+	-	-	-	-
Static image	+	+	-	+	+
External visualization software	-	-	+	-	-
SBML import	+	+	+	+	+
SBML export	+	+	+	+	+
Text file import	+	-	+	+	-
MATLAB export	+	+	-	-	-
Linux	+	-	+	+	*
MacOS	+	+	+	-	*
Windows	-	+	-	+	*
Pathway import from relational database	+	-	-	-	+
Model verification	+	+	-	-	-
Automated gap filling	-	+	+	-	-

Notes: Feature comparison of programs for flux balance optimization. + and - indicate the existence and absence of the feature, respectively. * No installation is needed, since FAME is exclusively web-based software.

**Table 2:** Experimentally determined and predicted growth rates. Experimentally determined specific uptake rates and growth rates were obtained at about half maximal cell dry weight (CDW).

Amino Acid	Strain	Uptake Rate [mmol g_CDW_^−1^ h^−1^]	Growth Rate [h^−1^]	
			Experiment	Model
Alanine	WT	5.7	0.160	0.239
	Δ262	5.6	0.235	0.231
	*tdaE*	8.2	0.333	0.342
Phenylalanine	WT	1.4	0.102	0.159
	Δ262	1.4	0.170	0.161
	*tdaE*	1.1	0.120	0.116
Leucine	WT	0.9	0.063	0.062
	Δ262	1.4	0.104	0.103
	*tdaE*	1.5	0.123	0.114

**Table 3:** Predicted carbon flux distribution dependent on the carbon source. Listed are the theoretical carbon flux distributions in % of the available carbon. FBAs were performed with experimentally determined specific uptake rates and growth rates (Table 2).

Amino Acid	Strain	% Cell Dry Weight	% CO_2_	% TDA
Alanine	WT	34.1	51.1	14.8
	Δ262	51.0	49.0	0
	*tdaE*	52.5	47.5	0
Phenylalanine	WT	31.6	49.5	18.9
	Δ262	49.5	50.5	0
	*tdaE*	49.6	50.4	0
Leucine	WT	45.5	54.5	0
	Δ262	51.0	49.0	0
	*tdaE*	50.7	49.3	0

## Data Availability

The metabolic models used in this study are available online. The model iMG481 is available at: mmtb.brenda-enzymes.org/models/view/11 (accessed on 9 Febraury 2021). The model iPin571 is available at: mmtb.brenda-enzymes.org/models/view/12 (accessed on 9 Febraury 2021). Source code of the Metano software toolbox is available at GitHub: github.com/JuliaHelmecke/metano (accessed on 9 Febraury 2021). A Docker image is available at Docker Hub: hub.docker.com/r/jhelmecke/metano (accessed on 9 Febraury 2021). A detailed documentation can be found at the project website: mmtb.brenda-enzymes.org/metano/manual (accessed on 9 Febraury 2021).

## Supplementary Materials

The following are available online at https://www.mdpi.com/2218-1989/11/2/113/s1, Table S1: The metabolic model of *Phaeobacter inhibens* DSM 17395 with integrated annotation, Table S2: Biomass composition for the wild-type, Δ262-kb plasmid-cured and *tdaE* transposon mutant strain for cells growing with l-alanine, l-phenylalanine or l-leucine as sole carbon source, respectively, Table S3: Protein composition of *Phaeobacter inhibens* DSM 17395, Table S4: Growth characteristics of the wild-type, Δ262-kb plasmid-cured and *tdaE* transposon mutant strain with l-alanine, l-phenylalanine and l-leucine as sole carbon source. Appendix: The detailed documentation of Metano, including use instructions, algorithms, and examples.
