# Supplementary material for: The Metano Modeling Toolbox MMTB: An Intuitive, Web-Based Toolbox Introduced by Two Use Cases
Source: Metabolites. 2021 Feb 17;11(2):113. doi: 10.3390/metabo11020113 (PMC7923039; doi:10.3390/metabo11020113)
Supplement: Supplementary file 1 [file metabolites-11-00113-s001.zip › Appendix-1.pdf]

Metano Toolbox

Documentation

February 8, 2021

1 Content

- Introduction
- Example model
- File formats
- Flux Balance Analysis
- Detection of dead ends
- Flux Variability Analysis
- Minimization of Metabolic Adjustment
- Knockout analysis
- Split-ratio Analysis
- Metabolite Flux Minimization
- SBML Support
- Other metano tools

2 Introduction

Metano is an open-source software toolbox for analyzing the capabilities of metabolic networks and for assisting in metabolic reconstruction. The software is written in Python and is designed to be easy to use and run reasonably fast. Metano contains efficient implementations of the established computational methods flux balance analysis (FBA), minimization of metabolic adjustment (MOMA), and flux variability analysis (FVA), as well as a script for automatically analyzing all specified single- or multi-gene knockouts using MOMA or FBA, respectively. In addition, Metano provides several newly developed methods, including the automated plausibility checking of metabolic models and methods for the metabolite-centric analysis of flux distributions: split-ratio analysis (SRA) and metabolite flux minimization (MFM).

All methods implemented in Metano are available both as Python functions and as stand-alone command line scripts. This page only describes the stand-alone programs, which take lists of reactions in plain ASCII format as input. Metano is designed to run in any Linux environment. A usage hint for any script in Metano can be obtained by launching the program with the command line option `-h`.

3 Example model

An *E. coli* core model can be downloaded in the SBML format from

`http://systemsbiology.ucsd.edu/Downloads/EcoliCore`.

The model can be converted to the Metano format via the command

```
sbmlparser.py -f ecoli_core_model.xml -o reactions_Ec_core.txt -p
    scenario_Ec_core.txt -q flux_Ec_core.txt -b _b$ -n -s
```

This produces the reaction file `reactions_Ec_core.txt`, the scenario file `scenario_Ec_core.txt`, and the flux file `flux_Ec_core.txt` in Metano's output file format. These files can be used as test input for trying out the programs in Metano.

4 File formats

In Metano, a metabolic network is represented by a pair of ASCII files: a reaction file describing the network itself and a scenario file containing the simulation parameters. The reaction file contains a list of reactions, including transporters and exchange reactions, which transport metabolites over the system boundary into or out of the system. The scenario file contains the definitions of inequality constraints, including lower and upper bounds for the reaction fluxes. It also contains parameters for the optimization methods, most importantly the definition of the objective function to be optimized and the solver to be used for optimization.

Both reaction and scenario files can contain comments. A comment starts with a hash character (#) and ends at the end of the line on which it occurs.

We published a syntax highlighting package for Visual Studio Code: `juliahelmecke.metano`

5 Reaction file

The reaction file contains lines of the form

```
<reaction name> : <substrates> <arrow> <products>
```

where `<substrates>` and `<products>` are lists of compounds (metabolites) given as

```
[coefficient] <compound name>
```

entries, which are separated by plus signs (+). If the stoichiometric coefficient is omitted, the default value of 1 is assumed. `<arrow>` is `<=>`, `-->`, or `<--` and specifies a constraint on the direction of the reaction. `-->` means that the reaction is irreversible and can only proceed from left to right, while `<--` means that it can only proceed from right to left. `<=>` means that the reaction is reversible. The flux through an irreversible reaction is implicitly constrained to be positive (`-->`) or negative (`<--`), respectively.

Example:

```
PBG-D : 4 porphobilinogen + h2o <=> hydroxymethylbilane + 4 ammonia
```

Transporters are defined as ordinary reactions that convert between a compound in one compartment and the same compound in another compartment. For distinguishing between metabolite pools in different compartments, compartment identifiers should be appended to the compound names. Example:

```
co2-trans : co2[c] --> co2[e]
```

Boundary fluxes are defined by reactions that are not in mass balance, i.e. they convert a compound to nothing or produce it from nothing. Example:

```
co2-boundary : co2[e] <=>
```

Reaction and compound names may contain any character except for whitespace characters (space, tab, newline), colon (:), hash (#), and the reserved arrow sequences (`<=>`, `-->`, `<--`).

6 Scenario file

The scenario file can contain five different types of parameter definitions:

1. Objective function for FBA
2. Flux bounds
3. Linear equality or inequality constraints
4. Solver to be used for optimization problems
5. Number of starting points (used only for nonlinear optimization)

The **objective function** and the sign of optimization (minimization or maximization) to be used in flux balance analysis is defined by a line of the form

```
OBJ <MAX|MIN> <objective function>
```

`<objective function>` is either a linear combination of reaction fluxes or a term of the form `PER_FLUX(<reaction name>)`. In the first case, the value of the linear function is minimized or maximized, respectively. In the second case, the nonlinear objective function "flux through single reaction divided by summed absolute flux over all reactions" is used. There may be only one OBJ definition per file.

In a linear combination of flux variables, reaction fluxes are identified by the corresponding reaction names as defined in the reaction file. For instance,

```
R1 - R2b + 2.5 R3
```

represents the expression "flux through reaction R1 minus flux through R2b plus 2.5 times flux through R3". As + and − can be part of reaction names, whitespace is required between operators, coefficients, and reaction names.

Flux bounds are defined by lines of the form

```
LB <reaction name> <value>
```

or

```
UB <reaction name> <value>
```

respectively. UB defines an upper bound, while LB defines a lower bound. `UB GLU-1 22` means that the flux through reaction GLU-1 is restricted to values less than 22. It is possible to restrict the flux through a reaction to a fixed value by setting the lower and upper bounds for that reaction to the same value. Gene deletions can be modeled by restricting to zero the fluxes through all reactions catalyzed by the enzyme to be knocked out.

Arbitrary **linear constraints** can be defined as equalities or inequalities of the form

```
<linear combination of fluxes> <sign> <value>
```

where `<sign>` is $<$, $>$, or $=$, and `<linear combination of fluxes>` is a linear combination of flux variables, as described above. For example, the constraint

```
R_complex-1A - R_complex-1B = 0
```

states that the reactions `R_complex-1A` and `R_complex-1B` must have the same flux value.

The syntax of linear constraints can also be used to define flux bounds. The lines

```
UB GLU-1 22
```

and

```
GLU-1 < 22
```

are treated as equivalent.

The **solver** to be used for solving optimization problems is specified by name in a line of the form

```
SOLVER <name>
```

The following solvers are currently available:

- `glpk` (default, recommended) and `lpsolve` for linear programming
- `cvxopt_qp` for quadratic programming
- `algencan` for nonlinear programming

If the given solver is not applicable to the optimization problem under consideration, the corresponding program will either emit an error message or use an applicable default solver and emit a warning. There may be at most one SOLVER definition per file.

The **number of random starting points** to try in nonlinear optimization can be specified by a line of the form

```
NUM_ITER <value>
```

There may be only one NUM_ITER definition per file.

7 Flux Balance Analysis

The script **fba.py** performs flux balance analysis (FBA) on a given metabolic network, i.e. it solves the following optimization problem:

> *Minimize/Maximize the given objective function in steady state under the given additional linear equality and inequality constraints with all flux variables constrained to the given bounds.*

The script is called as follows:

```
fba.py -r <reaction file> -p <scenario file> -o <output file> [-m]
```

It parses the given reaction and scenario files, formulates the optimization problem, applies the given solver to the problem, and writes the output to the given file. The optional command line switch `-m` instructs the program to return the optimal solution with the lowest total flux. As this solution is determined using binary search with one FBA at each step, this has a much longer running time than an ordinary FBA run.

The output is in form of a table with one entry for each reaction, listing the reaction name, the predicted flux value, and the effective lower and upper flux bounds. Like all programs in Metano, `fba.py` supports the command line option `-h`, which prints a help message regarding the usage of the script.

8 Detection of dead ends

deadends.py identifies *dead ends* in the metabolic network, i.e. compounds that cannot be produced and consumed in separate reactions. The usage is

```
deadends.py -r <reaction file> -p <scenario file> [-a] [-t <regex>]
```

Owing to the steady state condition, any reaction that contains a dead end is assigned a flux of zero in the FBA. The optional command line switch `-a` instructs the program to recursively identify all reactions that are blocked due to dead ends and all secondary dead ends induced by blocked reactions. Dead ends can indicate errors in the model, such as missing reactions, wrong reaction directions, and use of different names for the same compound.

The program can be instructed to temporarily leave the fluxes through transport and exchange reactions unconstrained. This allows distinguishing between dead ends resulting from the network topology and those induced by the virtual medium composition. To this end, a regular expression identifying the transport and/or exchange reactions can be passed to the program using the option `-t`.

The output, which is sent to the console, consists of a list of all dead-end metabolites followed by a list of all blocked reactions.

9 Flux Variability Analysis

The script **fva.py** performs flux variability analysis (FVA) on a given metabolic network. Its usage is

```
fva.py -r <reaction file> -p <scenario file> -o <output file>
    [-t <threshold>] [-s <solution file>]
```

It first performs a standard FBA (or reads in an FBA solution given via command line option `-s`) and then performs variability analysis by the following algorithm:

1. Introduce a new constraint: Original objective function must have a value of at least `<threshold>` (default: 0.95) times the optimal value (as determined from the original FBA).
2. Perform linear programming for each flux variable: Successively maximize and minimize each of the flux variables.

This way, the script determines bounds for the (sub)optimal solution space in all dimensions. The difference between the computed maximum and minimum for a flux variable is a measure for how strongly that flux is restricted by the additional constraint of (sub)optimality. The optimality threshold can be changed from .95 (= 95%) to any other value between 0 and 1 via the command line option `-t`.

The output is in form of a table with one entry for each reaction, listing the reaction name, the minimum flux value, the flux value in the FBA solution, the maximum flux value, the difference between maximum and minimum value, and the effective lower and upper flux bounds.

10 Minimization of Metabolic Adjustment

The script **moma.py** performs minimization of metabolic adjustment (MOMA) on the metabolic network and inequality constraints of a virtual mutant against the given FBA solution for the corresponding wild type, i.e. it solves the following optimization problem:

> *Minimize the squared Euclidean distance between the flux vector and the wild-type flux distribution in steady state under the given additional linear equality and inequality constraints with all flux variables constrained to the given bounds.*

The script is called as follows:

```
moma.py -r <reaction file> -p <scenario file>
    -w <wild-type solution file> -o <output file> [-s <solver>]
    [-i <num_iter>]
```

It parses the given reaction, scenario, and FBA solution files, formulates the quadratic optimization problem, applies the optionally given solver to the problem, and writes the output to the given file. If the command line option `-s` is omitted, solver `cvxopt_qp` is used by default. If a nonlinear solver is used, the number of random starting points to be used can be specified using the command line option `-i`.

Mutant and wild type must have the same reactions. Gene deletions can be modeled by restricting to zero the fluxes through all reactions catalyzed by the enzyme to be knocked out. MOMA output has the same format as FBA output (see above).

11 Knockout analysis

The script **knockout.py** performs an automated analysis of the virtual knockout mutants given as a list of reaction groups to be knocked out together. This is done by sequentially setting the fluxes through all reactions in each group to zero and performing FBA or MOMA against the wild-type solution. The usage is

```
knockout.py -r <reaction file> -p <scenario file> -o <output file>
    [-f <solution file prefix>] [-g <knockout group file>] [-n]
    [-m <always|never|auto>] [-w <wild-type solution>] [-s <solver>]
    [-i <num_iter>]
```

Reaction groups are defined using the command line option -g. <knockout group file> contains lines of the form

```
group_name;reaction_name
```

All lines with the same group_name define one reaction group. A reaction may belong to more than one reaction group. If the command line switch -n is present, all reactions not belonging to any group are knocked out individually (usually not desirable). If the option -g is omitted, all single-reaction "knockouts" are generated.

At first, the script performs an FBA to get the optimal wild-type flux distribution or reads it from file if the -w option is used. It then sequentially generates virtual knockout mutants for each reaction group and computes a flux distribution for each such "mutant". If the command line option -f is used, all generated flux distributions are written to files named <solution file prefix><group_name>.txt. The command line option -m selects the computational method:

- -m auto (default): Perform FBA first to determine viability, then perform MOMA only if the knockout has not been found to be lethal.
- -m always: Always perform MOMA (slower).
- -m never: Perform FBA only (fast, but less accurate).

If the command line option -s is omitted, solver cvxopt_qp is used by default. If a nonlinear solver is used, the number of random starting points to be used can be specified using the command line option -i.

The output is in form of a table with one entry for each reaction group, listing the group name, the percentage of the FBA objective function value obtained for the mutant in comparison to the wild type, the distance as measured by the actual MOMA objective function (Euclidean distance or squared distance, possibly multiplied by a constant factor), the absolute distance, and the FBA objective function value for the mutant.

Because of its long running time, this program has been parallelized using MPI, which allows it to be executed on computer clusters. On most MPI installations, a multi-process run of knockout.py can be launched by typing

```
mpiexec -n <number of processes> knockout.py <options>
```

in the console, provided that the MPI daemon is running.

Alternatively, FBA can be used for knockout analysis instead of MOMA. This is selected via the command line switch -v. In this case, the running time is much lower, but results may be less biologically meaningful.

12 Split-ratio Analysis

The script **splitratios.py** performs split-ratio analysis (SRA) on a given FBA solution for a given metabolic network. Its usage is

```
splitratios.py <solution file> -r <reaction file> -o <output file>
    [-u] [-t <threshold>]
```

This computes the split ratios for all metabolite nodes in the network. The command line switch `-u` excludes all metabolite nodes that have only one incoming and one outgoing flux (i.e. those lying on unbranched paths). The option `-t` further allows the exclusion of all metabolite nodes with a total flux below the given threshold.

The script can also be used to compute only the split ratios for a single metabolite. In this case the usage is

```
splitratios.py <solution file> -r <reaction file> -m <metabolite
    name>
```

This leads to a much clearer output than the long table produced when the command line option `-o` is used.

13 Metabolite Flux Minimization

The script **mfm.py** performs metabolite flux minimization (MFM) on a given metabolic network, i.e. it successively minimizes the flux through each metabolite node. Its usage is

```
mfm.py -r <reaction file> -p <scenario file> -o <output file>
    [-t <threshold>]
```

It first performs a standard FBA and then performs variability analysis by the following algorithm:

1. Introduce a new constraint: Original objective function must have a value of at least `<threshold>` (default: 0.95) times the optimal value (as determined from the original FBA).
2. Perform linear programming for each metabolite flux: Successively minimize each metabolite flux.

The output is in form of a two-column table with one entry for each metabolite, listing the metabolite name and the minimum flux value. The optimality threshold can be changed from .95 (= 95%) to any other value between 0 and 1 via the command line option `-t`.

14 Automated plausibility checking of metabolic models

The script **modelassert.py** checks a number of assertions (Boolean expressions that evaluate to `True` for the expected case) about a model's predictions and reports those that are violated. The assertion monitor is used as follows:

```
modelassert.py -r <reaction file> -p <scenario file>
    -a <assertion file> [-t <threshold>]
```

It reads in the given reaction file and scenario file, performs FBA, FVA, and split-ratio analysis, and checks the results against the list of assertions from the given file. All assertions that do not hold are reported.

Each line of the assertion file is one Boolean expression. Empty lines are allowed. Comments, which start with the hash sign (#) and end at the end of the line, are ignored. The following symbols can be used in assertions:

- `<reaction name>` – flux through reaction in FBA solution
- `min(<reaction name>)` – FVA minimum of flux through reaction
- `max(<reaction name>)` – FVA maximum of flux through reaction
- `<metabolite name>` – total flux going through metabolite in FBA solution
- `inratio(<metabolite name>, <reaction name>)` – fraction of metabolite produced via reaction in FBA solution
- `outratio(<metabolite name>, <reaction name>)` – fraction of metabolite consumed via reaction in FBA solution

Warning: This script must never be used in a non-safe environment (such as in a server-based application), because the Python `eval()` function is used to evaluate the assertions. It is the user's responsibility to use only Python functions that do not produce disruptive side effects.

15 SBML Parser

With **sbmlparser.py**, Metano includes a parser for models in the Systems Biology Markup Language (SBML). The parser reads an SBML file and writes a set of files in the native formats of Metano. The usage is as follows:

```
sbmlparser.py -f <SBML file> -o <r.filename> -p <s.filename>
    [-q <f.filename>] [-i <inf-level>] [-n] [-s] [-x <solver>]
    [-b <boundary regex>]
```

The parser reads the given SBML file and writes the reactions to the file designated by `r.filename`. The flux bounds are written to the scenario file `s.filename`. If the command line option `-q` is present, reaction fluxes (if present in the SBML file) are

written to file `f.filename`. The file format for fluxes is the output file format of `fba.py` (see above). The reaction file, scenario file, and flux file are overwritten if they exist.

Option `-i` can be used to specify an infinity level above which flux bounds are considered to be infinite (default: 1000). The same value is used for positive and negative infinity. The command line switch `-n` instructs the parser to use names in place of IDs for reactions and compounds, which can improve the readability of the resulting files. The switch `-s` suppresses the transfer of comments, which may clutter the reaction file. Option `-x` can be used to pass a solver to be written to the scenario file.

"Boundary species", which can be exchanged freely with the environment, are usually marked in SBML by having their `boundaryCondition` set to `"true"`. However, in some models this information is only encoded in the name of the compound (e.g. by the suffix `_b`). The identifying tag can be passed to the program as a regular expression via command line option `-b`. If boundary metabolites are not correctly identified, the model will not work in Metano FBA.

Error, warning, and info messages are written to the console. See above for a usage example.

16 SBML Writer

sbmlwriter.py converts a set of one reaction file, one scenario file, and (optionally) one flux distribution (i.e. FBA or MOMA solution file) to an SBML file. Its usage is

```
sbmlwriter.py -r <reaction file> -p <scenario file> -o <SBML file>
    [-q <flux file>] [-c <compartment regex>]
    [-d <default compartment>] [-i <inf-level>] [-s] [-b <suffix>]
```

The SBML writer reads a reaction file, scenario file, and (optionally) flux file and writes the output to `<SBML file>`.

The command line option `-c` can be used to specify the encoding of compartment information in the names of the compounds in the reaction file. For instance, `"\[(\w+)\]$"` means that a word in brackets at the end of the compound name (such as `[Cytosol]` or `[Extracellular]`) identifies the compartment. The regular expression must contain exactly one group (in parentheses). The option `-d` is used to define the default compartment to which all compounds not matching the regular expression are assigned (default: `Cytosol`).

If the option `-i` is present, finite values are used for all flux bounds. The value `<inf-level>` is assigned to all unbounded fluxes (with appropriate sign). The command line switch `-s` can be used to suppress the transfer of comments. If the option `-b` is present, boundary species (marked with the given suffix) are added to all reactions lacking either products or reactants.

17 Other metano tools

Metano provides a number of utilities that perform such tasks as converting files, extracting data from output files, and comparing output files. All these scripts support the command line option `-h`, which prints a help message regarding the usage of the script.

to_scatterplot.py This script allows to compare two flux distributions in a logarithmic scatter plot. The x and y files can be either flux distributions obtained from FBA, MOMA, FVA, or MFM analysis, or plain scenario files. The script reads the files or performs analyses depending on the given analysis method. The files that are passed to x and y must be of the same kind. If two scenario files are passed to the script, a reaction file must be provided (`-r`).

The usage is

```
to_scatterplot.py -x <x FILE> -y <y FILE> -m <fba|moma|fva|mfm|mva>
    [-r <reaction file> -c <config file> -o <output file> -b <obj
    fun>]
```

If an output file is given (`-o`, the scatter plot is saved in a file. Otherwise, an interactive scatterplot is shown.

The user can pass the name of the objective function to the `-b` flag of the script. By doing this, all differences between both scenarios that are only based on the change in objective function, are considered to be not significant.

It is possible to pass a configuration file, that is used to alter the style of the scatter plot, to the `-c` flag. This style file may contain the following parameters:

- `label_length = 15` (Maximum label length longer labels will be abbreviated)
- `ids_in_names = False` (True if BRENDA, KEGG, or MetaCyc IDs are in reaction names)
- `unique_names = False` (If True, keep names unique if reaction names are abbreviated)
- `min_value = 0.0` (Lower cutoff value for the plot)
- `insignificant_color = #545454` (Color for insignificant data points)
- `significant_color = #BE1E3C` (Color for insignificant data points)
- `xlabel = x file name` (label for the x axis; if omitted, the filename is used)
- `ylabel = y file name` (label for the y axis; if omitted, the filename is used)
- `title =` (Title for the plot; leave empty to disable the title)
- `show_errorbar = significant` (choose which errorbar to show [all, significant, none], works only for FVA)/MVA
- `show_labels = significant` (choose which point labels to show [all, significant, none])
- `arrowcolor = #BE1E3C` (color for label arrows, only works with textAlign)

- `labelcolor = #BE1E3C` (color for point label text; does not work with textAlign)
- `significance_threshold = 0.1` (threshold for significance calculation)
- `fontsize = 10` (general fontsize)
- `figsize = 10 8` (the figure size, x and y seperated by one space)
- `plotgrid = False` (show the grid of the plot)
- `arrow_line_width = 1` (the width of the label arrows; only works with textAlign)

If scenario files are passed to `-x` and `-y`, the metabolic variability analysis (MVA) kann be performed by setting `-m mva`. First of all, this method performs FBA and shows the result of split ratio analysis as data points. Secondly, a MFM for each scenario is performed, with both, metabolic flux minimization and maximization. The resulting flux range is shown as error bars in the scatterplot.

to_evaluate_objective.py This script improves debugging of Metano models by evaluating the production flux of each$ It iterates through all metabolites of the objective function and maximizes their production flux. The usage is

```
to_evaluate_objective.py -r <reaction file> -s <solution file>
```

The script prints its output in the console and lists all metabolites, that could not be produced by the metabo$

to_rea2m.py This script reads in a reaction file and exports the stoichiometric matrix corresponding to the metabolic network as a .m file, which can be read by MATLAB and GNU Octave. The usage is

```
to_rea2m.py -r <reaction file> -o <output file>
```

The output file should have the extension ".m".

to_list_active_metab.py This script reads in an FBA/MOMA solution file and the underlying reaction file and prints the names of all compounds present in reactions with a non-zero flux along with the number of such reactions in which they appear. The usage is

```
to_list_active_metab.py -r <reaction file> -s <solution file>
    [-c <cutoff>]
```

Reactions with a flux value below the given cutoff (default: 1E-10) are considered inactive. The output, which is sent to the console, consists of a list of all active metabolites, each with the number of active reactions it is involved in, followed by a list of all active reactions.

to_diff_solutions.py This script computes the absolute difference between two flux distributions given as FBA/MOMA solution files. The usage is

```
to_diff_solutions.py <file1> <file2> [-o <output file>] [-c <cutoff>]
```

The output is in form of a table with one entry for each reaction, listing the reaction name, the absolute flux difference, and the values of the flux in file1 and file2, respectively. If no output file is given, the output is sent to the console, and the latter two columns are omitted. The output is sorted by the absolute flux differences in descending order. Only flux differences above the given cutoff value are reported. If no cutoff is specified, the default value of 1E-10 is used.

to_check_constraints.py This script checks whether the given flux distribution (FBA/-MOMA solution file) conforms to the constraints imposed by the given reaction and/or scenario file. It lists all constraints violations. There is zero tolerance, so that e.g. a value of -1E-25 violates an “LB 0” constraint. The usage is

```
to_check_constraints.py <solution file> [-r <reaction file>]
    [-p <scenario file>]
```

The list of violated constraints is printed to the console.

to_fix_names.py This script reads in a reaction file that contains whitespace characters in the reaction or compound names and exports a fixed reaction file, in which these characters have been replaced with underscores (_). The usage is

```
to_fix_names.py -i <input file> -o <output file>
```

Whitespace characters (space, tab, newline) are normally illegal in compound names, but might still occur if the file has been automatically generated, e.g. from enzyme or pathway databases. This script fixes such files, so that the metabolic network defined therein can be analyzed with Metano.
