# Supplementary material for: The Metano Modeling Toolbox MMTB: An Intuitive, Web-Based Toolbox Introduced by Two Use Cases
Source: Metabolites. 2021 Feb 17;11(2):113. doi: 10.3390/metabo11020113 (PMC7923039; doi:10.3390/metabo11020113)
Supplement: Supplementary file 1 [file metabolites-11-00113-s001.zip › Tables-S2-S4.pdf]

Supplementary data

Table S2. Biomass composition for the wild-type, Δ262-kb plasmid-cured and *tdaE* transposon mutant strain for cells growing with L-alanine, L-phenylalanine or L-leucine as sole carbon source, respectively. Listed are mean values of 6 biological replicates for DNA, RNA, lipids, protein and polyhydroxybutyrate, respectively. DNA, RNA, protein and lipid content were quantified as previously described (Wolf et al. 2016). PHB was alkaline hydrolyzed and the monomers were quantified with the D-3-hydroxybutyric acid enzymatic assay of r biopharn (Darmstadt, Germany). Peptidoglycan content was estimated as previously described (Dannheim et al. 2017). All values are listed in % of biomass.

**Component**	**Alanine**			**Phenylalanine**			**Leucine**			**Reference**
	WT	Δ262	*tdaE*	WT	Δ262	*tdaE*	WT	Δ262	*tdaE*	
**DNA**	1.6 ± 0.1	1.4 ± 0.3	1.2 ± 0.2	1.3 ± 0.2	2.0 ± 0.3	1.1 ± 0.2	1.4 ± 0.2	1.6 ± 0.3	0.9 ± 0.3	this work
**RNA**	4.5 ± 0.3	6.8 ± 0.9	5.9 ± 0.5	5.4 ± 0.8	5.8 ± 0.3	3.9 ± 0.7	3.4 ± 0.3	4.3 ± 0.8	3.7 ± 0.1	this work
**Lipids**	6.8 ± 0.4	5.9 ± 0.5	5.3 ± 0.6	6.1 ± 0.6	5.3 ± 0.9	5.9 ± 0.5	5.6 ± 0.6	5.9 ± 0.7	3.4 ± 0.5	this work
**Protein**	54 ± 3	56 ± 3	59 ± 4	55 ± 3	52 ± 4	51 ± 4	53 ± 4	59 ± 5	50 ± 4	this work
**Polyhydroxybutyrate**	7 ± 2	7 ± 0.5	7 ± 1	13 ± 1	14 ± 3	24 ± 2	17 ± 2	14 ± 4	23 ± 3	this work
**Peptidoglycan**	12 ± 2	12 ± 2	12 ± 2	12 ± 2	12 ± 2	12 ± 2	12 ± 2	12 ± 2	12 ± 2	this work
**Lipopolysaccharide**	3.4	3.4	3.4	3.4	3.4	3.4	3.4	3.4	3.4	Feist et al 2007
**Glutamate**	4	4	4	4	4	4	4	4	4	this work
**Residual soluble pool**	2	2	2	2	2	2	2	2	2	
**Sum**	95 ± 8	99 ± 7	100 ± 8	102 ± 8	100 ± 11	107 ± 9	102 ± 9	106 ± 13	102 ± 10	

Table S3. Protein composition of *Phaeobacter inhibens* DSM 17395. Amino acids of cell hydrolysates were quantified by HPLC as previously described (Dannheim et al. 2017). Glutamate and alanine were corrected for the estimated amount in peptidoglycan (by quantification of *meso*-diaminopimelate). Glutamate was further corrected for determined intracellular amounts (4% of CDW). Asparagine and glutamine were estimated by their amino acid sequence ratio to aspartate and glutamate, respectively. Additionally, cysteine and tryptophan were estimated by their amino acid sequence ratio to tyrosine. These estimations are based on the assumption that every protein is present once.

Amino acid	mmol $g_{protein}^{-1}$	Amino acid	mmol $g_{protein}^{-1}$
**Alanine**	0.63 ± 0.08	**Leucine**	0.64 ± 0.03
**Arginine**	0.48 ± 0.03	**Lysine**	0.24 ± 0.03
**Asparagine**	0.70 ± 0.03	**Methionine**	0.12 ± 0.01
**Aspartate**	0.30 ± 0.01	**Phenylalanine**	0.32 ± 0.02
**Cysteine**	0.13 ± 0.00	**Proline**	0.54 ± 0.05
**Glutamate**	0.40 ± 0.08	**Serine**	0.49 ± 0.02
**Glutamine**	0.25 ± 0.05	**Threonine**	0.56 ± 0.02
**Glycine**	0.94 ± 0.02	**Tryptophan**	0.10 ± 0.00
**Histidine**	0.13 ± 0.01	**Tyrosine**	0.20 ± 0.01
**Isoleucine**	0.37 ± 0.02	**Valine**	0.56 ± 0.02

Table S4. Growth characteristics of the wild-type, Δ262-kb plasmid-cured and *tdaE* transposon mutant strain with L-alanine, L-phenylalanine and L-leucine as sole carbon source. Biomass yield ($Y_{X/C}$) was determined by determination of maximal CDW values and total consumed carbon. Maximal growth rates μ_{max} were determined by linear fit of the logarithmized CDW values. *CDW_{max} for *tdaE* on L-alanine was estimated due to missing data point.

Amino acid	Strain	CDW_{max} [g L^{-1}]	$mmol_C$ consumed	$Y_{X/C}$ [g mol_C^{-1}]	μ_{max} [h^{-1}]
	WT	0.72 ± 0.08	82 ± 6	8.8 ± 1.1	0.217 ± 0.002
**Alanine**	Δ262	1.26 ± 0.01	84 ± 0.3	15.0 ± 0.1	0.269 ± 0.001
	tdaE	1.1* ± 0.00	88 ± 1	12.5 ± 0.1	0.350 ± 0.003
	WT	0.78 ± 0.05	102 ± 3	7.7 ± 0.5	0.099 ± 0.003
**Phenylalanine**	Δ262	1.32 ± 0.02	108 ± 3	12.2 ± 0.4	0.216 ± 0.002
	tdaE	1.16 ± 0.04	110 ± 1	10.6 ± 0.3	0.147 ± 0.002
	WT	1.12 ± 0.02	100 ± 5	11.2 ± 0.6	0.100 ± 0.003
**Leucine**	Δ262	1.26 ± 0.02	84 ± 2	15.0 ± 0.5	0.110 ± 0.005
	tdaE	1.29 ± 0.03	89 ± 2	14.5 ± 0.4	0.123 ± 0.001
